# Increased incidence of glaucoma medication usage in middle-aged Australian males taking antiretroviral medication – a population-based study

**DOI:** 10.1186/s12348-020-00218-y

**Published:** 2020-11-03

**Authors:** Wen-Shen Lee, Shaun Parsons, Dean Cugley, Sophie Rogers, Lyndell L. Lim, Anthony Hall

**Affiliations:** 1 Ophthalmology, The Alfred Hospital, Alfred Health, Melbourne, Australia; 2 Ophthalmology, Royal Victorian Eye and Ear Hospital, Melbourne, Australia; 3 Centre for Eye Research Australia, Royal Victorian Eye and Ear Hospital, Melbourne, Australia; 4 Ophthalmology, Department of Surgery, University of Melbourne, Melbourne, Australia

**Keywords:** Glaucoma, Human immunodeficiency virus, Acquired immune deficiency syndrome, Antiretroviral, Intraocular pressure, Glaucomatous optic neuropathy, Ocular hypertension

## Abstract

**Background:**

To investigate a possible association between glaucoma and the use of anti-retroviral therapy (ART) for HIV in the Australian population.

**Methods:**

A retrospective review of Australian Pharmaceutical Benefits Scheme data was undertaken from July 2012 to December 2016, inclusive. Three patient groups were compared: those on both topical intraocular pressure (IOP) -lowering medication and ART, those on ART only, and those on IOP-lowering medication only, using the 2016 Australian resident population to estimate prevalence. Odds ratios (95% confidence intervals, [CI]) with Fishers exact test for *p* values were calculated stratified by age and gender.

**Results:**

The number of prescriptions for topical glaucoma medications in the general Australian population increased progressively by age with a peak prevalence in those aged 80 years and above. Prevalence of ART was highest in males aged 40–49 and 50–59 years (0.41% [CI 0.40, 0.42] and 0.44% [CI 0.43, 0.45], respectively). Our analysis identified an increase in the prescription of IOP-lowering medication in males on ART aged 30–39 (OR 2.23 [CI 1.32, 3.75], *p* = 0.007) and 40–49 (OR 1.86 [CI 1.42, 2.43], *p* < 0.001), compared to those not on ART. There were no statistically significant increased odds for females or males aged 50 years or more.

**Conclusion:**

Compared with the known increase in glaucoma prevalence with age in the general Australian population, a statistically significant increased prevalence in use of IOP-lowering medications was found in males on ART aged 30–49 years. The mechanism for this is yet to be determined, but possible causes include sequelae of HIV infection, a drug-induced side effect, or increased medical surveillance.

## Background

Glaucoma is a group of optic neuropathies characterised by degeneration of retinal ganglion cells leading to the loss of optic nerve axons and vision [1]. It affects approximately 70 million people worldwide and is the leading cause of permanent blindness worldwide, with approximately 10% of patients blinded bilaterally by the disease [2, 3]. In Australia, it is estimated that approximately 2.5% of the population (150,000 people) above the age of 40 have glaucoma, and approximately 5% (275,000) have ocular hypertension [4, 5]. The incidence of glaucoma increases exponentially with age [6], with approximately 5 to 8% of patients aged 75 to 85 being diagnosed with glaucoma [7, 8]. Diagnosis of glaucoma is based on morphologic, structural and functional analysis of the optic nerve head (ONH) and retinal nerve fibre layer (RNFL). Tests used to help make the diagnosis include tonometry, visual field analysis and optical coherence tomography (OCT) [9]. The mainstay of treatment is topical therapy to lower intraocular pressure (IOP), with various other laser and surgical options available to manage the disease [10].

Human Immunodeficiency Virus infection is characterised by a depletion of cell-mediated immunity via progressive loss of CD4+ T cells, leading to increased risk of opportunistic infections and the Acquired Immunodeficiency Syndrome (AIDS) [11]. Approximately 36.9 million people are affected by HIV worldwide [12]. Notably, the incidence of new HIV infections is decreasing worldwide, having peaked in 1997, while the prevalence is steadily increasing by a rate of approximately 1.2% per year since 2000 [13]. Australia has a low prevalence of HIV, with approximately 0.14% of the population infected and a stable incidence of approximately 1000 new cases annually from 2007 to 2018 [14]. The mainstay of HIV treatment is antiretroviral therapy (ART). Historically, ART was initiated in patients with a CD4 count of below 350 or those diagnosed with an AIDS-defining illness [15–17]. In 2015 however, the START and TEMPRANO trials were published, leading to a change in practice where all HIV positive patients were commenced on ART from the time of diagnosis [18, 19].

HIV infection is associated with various ocular manifestations, including HIV retinopathy, CMV retinitis, uveitis, vascular abnormalities, neoplasias, neuroretinal disorders and other ocular complications which are mainly secondary to immunodeficiency but may also be due to primary HIV infection itself [20]. HIV-neuroretinal disorder affects approximately 16% of patients with AIDS and is characterised by decreased contrast sensitivity, colour vision, visual field anomalies, thinner RNFL layers and poorer electrophysiological responses [21, 22], (including poorer foveal and conventional full field pattern-shift visual evoked potentials, f-VEPs and c-VEPs). This is presumed to be a direct consequence of HIV infection rather than due to secondary opportunistic infection [23]. HIV infection is also associated with more general neurodegenerative disease [24, 25].

While HIV optic neuropathy is a well-documented entity, there is little literature on the incidence or prevalence of glaucoma within the HIV-positive population. It is possible that HIV-neuroretinal disorder may mimic glaucoma and either mask the diagnosis of glaucoma or alternatively lead to an increased rate of diagnosis of glaucoma.

The Longitudinal Study of Ocular Complications of AIDS (LSOCA) study by Jabs et al. in 2007 reported decreased contrast sensitivity, Goldman perimetry results as well as Humphrey Visual Field mean deviation indices in patients with AIDS [26, 27], without any measurement of IOP or comment on the incidence or prevalence of glaucoma. After an informal review of our local patient cohort, we noted an apparent disproportionately higher rate of glaucoma medication prescriptions in patients on ART. This raises concern that patients with HIV or AIDS may be at increased risk of glaucoma (or a glaucoma like degenerative optic neuropathy). With a high incidence of both HIV and glaucoma worldwide, any link between HIV and glaucoma could have implications for screening protocols. Our study aims to investigate the potential link between HIV and glaucoma.

## Methods

The purpose of our study was to find a possible correlation between glaucoma and use of ART, especially in patients with advanced disease. To achieve this, we utilised the Australian Pharmaceutical Benefits Scheme (PBS) and retrospectively reviewed the records from July 2012 to December 2016 inclusive.

PBS is a government-subsidised program for holders of a Medicare card, substantially reducing the cost of medications for card holders [28]. All Australian citizens and permanent residents are eligible for the PBS and uptake is nearly universal. Each medication has a unique code, therefore allowing numbers and prescriptions filled to be tracked according to these codes. This allows for medication dispensing to be audited and tracked [28]. Accessing the PBS thus allowed us to acquire the total number of patients on medication for HIV, glaucoma or both.

Using the PBS database, three patient groups were identified. The first group were patients on ART and no glaucoma treatment. The second group consisted of patients on topical IOP-lowering medication without ART. The third group consisted of patients on both topical IOP-lowering medications and ART. Each group was analysed according to age groups (see table) and sex to allow comparison. Ultimately, we calculated the rate of IOP-lowering medication usage in patients taking ART, relative to the rest of the Australian population (not taking ART). Using PBS codes, the number of patients taking individual ART medications by name and category (see Table 1) as well as IOP-lowering therapy by name and category (see Table 2) was determined. Patients taking combination therapy (for either glaucoma or ART) were identified under separate codes and included. The 2016 Australian resident population figures were sourced from the Australian Bureau of Statistics (ABS) and used to establish the prevalence of usage of each medication. Patients under the age of 10 were excluded from this study. Oral acetazolamide (Diamox) was not considered to be an IOP-lowering therapy for the purposes of this study as it is not indicated for chronic glaucoma management in Australia and may be used for other medical conditions and thus generate significant confounding. Statistical analysis was calculated via odds ratios and 95% confidence intervals, with Fisher’s exact test for *p*-values calculated for each age and gender strata. The study was approved by the External Request Evaluation Committee and the Department of Human Services (DHS), Australia. The datasets used and/or analysed during the study are available from the corresponding author on reasonable request.

**Table 1:** PBS list of HIV medications. RTI = Reverse transcriptase inhibitors

Protease inhibitor	Atazanavir	Darunavir	Fosamprenavir	Indinavir	Ritonavir	Saquinavir	Tipranavir
**Nucleoside & Nucleotide RTI**	Abacavir	Didanosine	Emtricitabine	Lamivudine	Stavudine	Tenofovir	Zidovudine
**Non-Nucleoside RTI**	Efavirenz	Etravirine	Nevirapine	Rilpivirine			
**Integrase Inhibitor**	Dolutegravir	Raltegravir					
**Fusion Inhibitor**	Enfuviritide						
**Entry Inhibitor**	Maraviroc

**Table 2:** PBS list of glaucoma medications

Drug class				
**Prostaglandin Analogues**	Bimatoprost	Latanoprost	Tafluprost	Travoprost
**Beta blockers**	Timolol	Betaxolol		
**Sympathomimetics**	Apraclonidine	Brimonidine		
**Carbonic Anhydrase Inhibitors**	Brinzolamide	Dorzolamide		
**Parasympathomimetics**	Pilocarpine			

## Results

Australia’s resident population in 2016 consisted of 10,399,578 males and 10,670,041 females. A total of 22,914 males and 5614 females were prescribed ART. 194,388 males and 225,309 females were prescribed IOP-lowering therapy. A total of 367 males and 67 females were prescribed a combination of ART and IOP-lowering therapy.

**Fig. 1:**
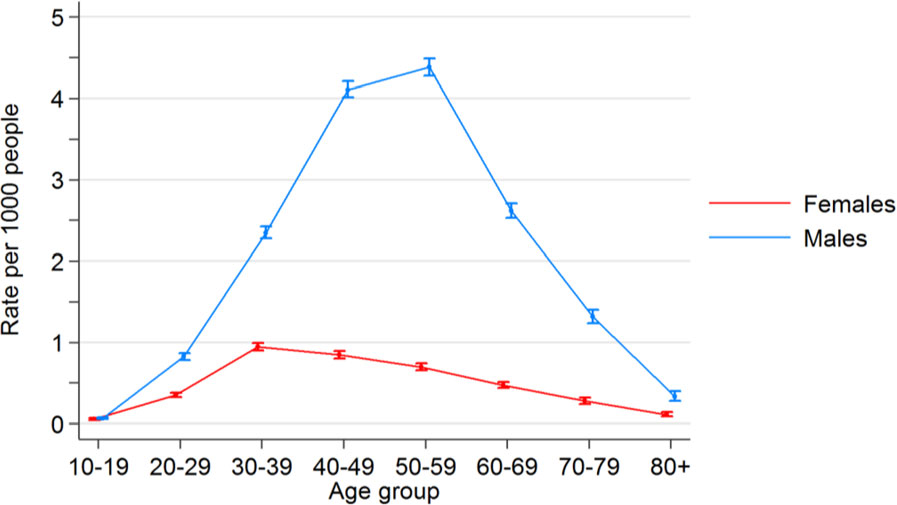
Rate of anti-HIV medication use in the 2015 Australian resident population

**Fig. 2:**
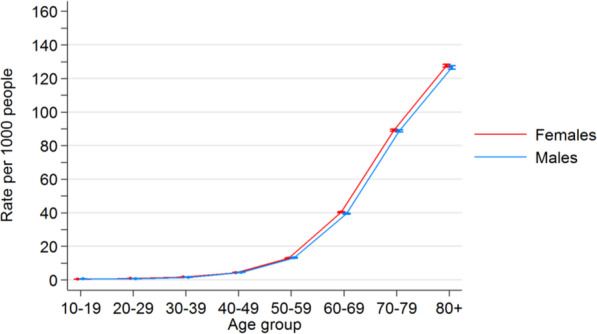
Rates of anti-Glaucoma medication use in the 2016 Australian resident population

The PBS-listed HIV medications are shown in Table 1. Including combination therapy, a total of 59 different codes were found and data pooled. PBS-listed IOP-lowering medications are shown in Table 2. Including combination therapy, a total of 53 codes were obtained and their data pooled. Figures 1 and 2 show graph plots depicting the incidence of medication use among age and sex groups. The rate of ART usage was highest among males aged 40–49 (0.41%, [CI 0.40, 0.42]) and 50–59 (0.44%, [CI 0.43, 0.45]), while the rate of IOP-lowering medication exponentially increased with age.

Crude rates for the period July 2012 to December 2016 inclusive were calculated. Filled shapes indicate the crude rate for that age group; error bars indicate 95% confidence intervals for the crude rate. The graphs show males having significantly higher rates of ART usage, especially in middle-aged males. Raw numbers can be found in the supplementary data section.

Crude rates were calculated for the period July 2012 to December 2016 inclusive. Filled shapes indicate the crude rate for that age group while error bars indicate 95% confidence intervals for the crude rate. The graph shows a significant increase in anti-glaucoma medication use with age. Raw numbers can be found in the supplementary data section.

The odds ratios were calculated for PBS data from July 2012 to December 2016 inclusive. Filled shapes indicate the OR for that age group while error bars indicate 95% confidence intervals for the OR. There was a statistically significant OR for males in the 30–39 and 40–49 age groups. Raw numbers can be found in Table 3.

**Table 3:** Rates of anti-HIV and anti-glaucoma medication by sex and age group in 2016 Australian residents

Gender	Age group	On Glaucoma Drops		No Glaucoma Drops		OR (95%CI)	P
		Anti-HIV meds [28]	No HIV meds [28]	Anti-HIV meds [28]	No HIV meds [29]		
Females	10 to 19	0	675	74	1,415,661	0.00 (0, 108.88)	1.000
	20 to 29	1	1493	606	1,737,137	1.92 (0.00, 10.89)	0.407
	30 to 39	6	2930	1606	1,704,755	2.17 (0.99, 4.75)	0.062
	40 to 49	6	7083	1377	1,631,798	1.00 (0.46, 2.19)	0.838
	50 to 59	21	20,001	1052	1,519,165	1.52 (0.99, 2.33)	0.077
	60 to 69	21	51,199	580	1,220,504	0.86 (0.56, 1.33)	0.603
	70 to 79	10	70,897	209	725,053	0.49 (0.26, 0.91)	0.023
	80 +	2	70,964	58	485,097	0.24 (0.00, 0.88)	0.030
Male	10 to 19	1	851	91	1,490,374	19.25 (0, 109.96)	0.051
	20 to 29	3	1321	1458	1,773,002	2.76 (0.94, 8.14)	0.097
	30 to 39	14	2678	3963	1,688,398	2.23 (1.32, 3.75)	0.007
	40 to 49	54	7070	6496	1,580,668	1.86 (1.42, 2.43)	< 0.001
	50 to 59	102	19,504	6416	1,462,098	1.19 (0.98, 1.45)	0.081
	60 to 69	125	48,284	3085	1,176,549	0.99 (0.83, 1.18)	0.928
	70 to 79	55	66,006	925	678,200	0.61 (0.47, 0.80)	< 0.001
	80 +	13	48,307	113	333,354	0.79 (0.45, 1.40)	0.504

**Fig. 3:**
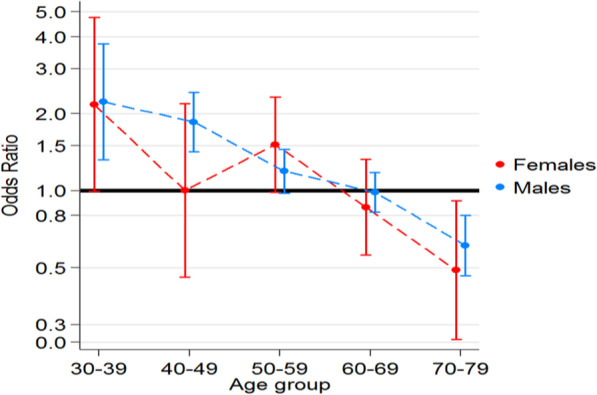
Odds ratios of anti-Glaucoma medication in anti-HIV prescription population compared to 2016 Australian population

Our study noted an odds ratio for Australian males in the 30–39 and 40–49 age groups, of 2.23 and 1.86 respectively (*P* < 0.05) on HIV medications to also be prescribed topical IOP-lowering drugs (See Fig. 3 and Table 3). The overall numbers were however low. The 30–39 age group had 14 males (*p* = 0.007) while the 40–49 age group had 54 males (*p* < 0.001). There was a trend for males aged 50–59 on HIV medications to also be prescribed topical IOP-lowering therapy (*n* = 102, OR 1.19, p 0.081). There was a trend for females aged 30–39 on HIV medications to also be prescribed topical IOP-lowering therapy (OR 2.17, p 0.06). However, the number in this group was noted to be even smaller (*n* = 6). Our study found a decreased OR for Australian males (*p* < 0.001) and females aged 70–79 (*p* = 0.023) as well as females aged 80 and above (*p* = 0.03) who were on ART to also be prescribed topical IOP-lowering therapy. Fifty-five males aged 70–79 were on ART and IOP-lowering medication at the same time (p < 0.001) while a small sample of 12 females aged 70+ were in this category (*p* < 0.05). Figure 3 illustrates the OR graph adjusted for age and sex groups.

Odds ratios and p-values were calculated for rates of anti-glaucoma medication use within the population taking medications for HIV.

## Discussion

We identified an increased OR of up to 2.23 for Australian males aged 30–49 on HIV medication to require IOP-lowering therapy. A similar trend was seen with females in this age group (*p* = 0.062), although the study population was likely too small to elucidate a statistically significant effect. We have several proposed explanations for why patients on ART appear to have a higher rate of usage of IOP-lowering therapy that we explore herein.

The first theory would be the possibility that HIV/AIDS causes glaucoma. While HIV and especially AIDS is associated with optic nerve dysfunction, glaucomatous injury to the optic nerve has never been described in the literature. Rather, HIV and a low CD4 count are ostensibly associated with hypotony and thickening of the RNFL in the superior and temporal quadrants [30]. Investigating HIV as a risk factor for the development of glaucoma in younger to middle-aged patients will require further research to establish a relationship between CD4 count and viral load with optic nerve function in order to validate this hypothesis.

There are, however, other explanations for why this patient group may have a higher incidence of being prescribed IOP-lowering medication. The first hypothesis would be HIV-associated microvasculopathy of the optic nerve. Microvascular changes of the optic nerve have been described, with ultrastructural changes including decreased axon volume, loss of pericytes, thickening of the basal lamina and narrowing of the lumina of retinal capillaries [31–33]. These changes could clinically and functionally mimic glaucomatous optic neuropathy, resulting in a misdiagnosis of glaucoma and prescription of IOP-lowering therapy.

A second possible reason for the increased incidence of IOP-lowering therapy being prescribed for Australian males aged 30–49 taking ART would be a drug-induced ocular hypertension or glaucoma. To investigate this postulation, the Monthly Index of Medical Specialities [34] online drug database was reviewed for ocular side effects of each individual anti-HIV drug (see Table 123) [34]. While there were no reports of “glaucoma” or “raised intraocular pressure”, we found that Ritonavir, a protease inhibitor (PI) has a reported rate of unspecified visual field defects in 2% or less in its published Prescriber Information [35]. Mitochondrial injury is a well-documented side effect of nucleoside reverse transcriptase inhibitors (NRTI) [36, 37], and is important in the pathogenesis of glaucoma [38]. While drug-induced mitochondrial toxicity is less pronounced in later generations of NRTIs it may still lead to an optic neuropathy and the prescription of glaucoma drops [39–41]. While plausible, the causal link between ART and glaucomatous optic neuropathy will require further investigation to elucidate.

A third possible mechanism for the increased OR for men aged 30–49 on ART to be concurrently taking IOP-lowering medication would be that RNFL changes observed in HIV neuro-retinal disorder being inadvertently misdiagnosed as glaucomatous optic neuropathy. The most common RNFL defects seen are often in the inferior portion of the disc, which can mimic glaucomatous changes [42]. Chronic inflammation in chronic HIV infection is associated with neurodegenerative disease [43]. This neurodegenerative disease may be mistaken for glaucoma. There is also evidence that chronic inflammation itself may be one of the pressure independent mechanisms of glaucoma [44]. If so, this may lead to a true increased incidence of glaucoma in HIV infected patients. It is also possible that given HIV patients are an actively monitored group, they are more likely to undergo regular ophthalmic assessments, including screening for infections including CMV retinitis, therefore allowing earlier diagnosis of glaucoma and leading to an apparent higher incidence of glaucoma than seen in the non-HIV controls.

Fourthly, the increased incidence of glaucoma medication use could be due to other ocular diseases linked to HIV/AIDS that may require IOP-lowering therapy. However, hypertensive uveitis in HIV/AIDS is uncommon, with the reported incidence of uveitis in HIV/AIDS ranging from 0.8 to 5.0% [45–47]. It is typically associated with viral infections such as varicella zoster, herpes simplex and CMV anterior uveitis [48, 49], as well as other infections such as toxoplasmosis and syphilis [50]. While HIV is a risk factor for the development of herpes zoster ophthalmicus (HZO) which can cause a hypertensive uveitis in up to 56% of HZO cases [51–56], the overall incidence of HZO in HIV/AIDS patients is low [57]. CMV is also known to cause a hypertensive anterior uveitis especially in immunocompetent Asian patients [58], although it is much more likely to manifest as retinitis in HIV/AIDS patients without need for IOP-lowering therapy [45, 59, 60]. Patients with CMV retinitis may however develop Immune Recovery Uveitis (IRU) after commencement of ART [61], which carries an approximately 33% incidence of ocular hypertension [62]. Although IRU is uncommon with a rate of 0.6–2.2 cases per 100 person years, it is an important cause of hypertensive uveitis in patients receiving ART [63]. Syphilis infection is common amongst HIV-positive patients [64, 65], and is also known to cause hypertensive anterior uveitis [66, 59]. Hypertensive uveitis must therefore be considered as a potential cause for at the high incidence of IOP-lowering therapy among patients on ART.

Our study noted that the incidence of HIV medication prescription and topical IOP-lowering therapy in the general population is consistent with the epidemiological profile of both diseases. The low incidence of ART usage in the elderly can be explained by the mortality rate of the disease in the 1980s and 1990s, leaving relatively few survivors in this age group. However, it is unclear as to why the proportion of patients on ART being prescribed glaucoma drops appears to decrease in older age groups. If HIV or ART causes glaucoma, the number of patients on ART requiring IOP-lowering therapy should remain proportionately high in older age groups. Neurodegenerative disease is an independent prognostic marker for mortality in HIV [67]. If glaucoma medication use in the HIV population is a marker of other neurodegenerative diseases then these patients may have a survival disadvantage and thus be underrepresented in the elderly HIV population.

### Study strengths and limitations

By using PBS data we were able to capture the majority of patients with HIV infection or glaucoma or both in the community. We identified 419,695 patients using glaucoma drops and 28,528 patients using ART. These large numbers enable us to detect small effects.

However, there are several limitations to our study. Firstly, PBS records did not give us information on the indication for the prescription of IOP-lowering therapy. We therefore do not know if the medications were prescribed for an actual diagnosis of glaucoma or for hypertensive uveitis or undifferentiated optic neuropathy. If IOP-lowering drops were prescribed for indications other than glaucoma, then we may have overestimated the incidence of glaucoma in our patients. Similarly, if patients developed glaucoma and were not prescribed topical treatment or were treated with laser or surgery then our methodology will have underestimated the incidence of glaucoma. Secondly, there has not been any measurement of IOP to link IOP rise with HIV medication use, or whether the incidence of IOP-lowering therapy correlates with viral load and CD4 count. Further studies will be required to establish causality. Thirdly, our study only included patients with HIV on ART. Based on 2016 data, patients not on ART represent about 15% of HIV positive individuals in Australia [14]. This is consistent with guideline changes in 2015 where universal treatment was recommended for all HIV-infected individuals. We are therefore unable to determine whether glaucoma related to HIV is a disease-related effect or a medication-induced one. This also raises the point about viral load and its correlation with optic neuropathy. Fourthly, medications were not analysed by subclass. At this point in time, we cannot point to any particular anti-HIV medication that may be responsible for the apparent glaucomatous ocular changes. Lastly, the significant findings were only found in the male group, without an equivalent significant finding in females. This is likely to be secondary to small sample size, given that there is no established gender predilection for open angle glaucoma [68].

### Directions for the future

While these findings are significant and reinforce the possibility that glaucoma or similar optic nerve disease may be associated with HIV/AIDS or its treatment. Further studies will be required to clarify these effects. A longitudinal study with IOP measurement and measurements of optic nerve function in HIV positive individuals would be useful. Future studies should also stratify HIV positive patients into those taking ART and those not on treatment, as well as analysing patients by viral load and CD4 count. Patients on ART should be analysed according to medical subgroup to determine whether a particular antiretroviral medication may be implicated in the development of glaucoma. Lastly, a larger study population with sample sizes matched for age and sex will help generate more significant findings.

## Conclusion

Our study shows that young patients receiving ART have a higher incidence of use of IOP lowering drops. This is most pronounced in males aged 30 to 49. While this raises suspicion that HIV may be associated with the development of glaucoma or similar optic nerve disease, further prospective studies with a larger sample size will be required to elucidate any causation, whether it be disease-related or medication-related, or whether HIV/AIDS causes ocular diseases that can mimic glaucoma.

## Supplementary information

**Additional file 1.**

## Data Availability

The datasets used and/or analysed during the current study are available from the corresponding author on reasonable request.

## Abbreviations

HIV: Human immunodeficiency virus
AIDS: Acquired immune deficiency syndrome
ART: Antiretroviral therapy
IOP: Intraocular pressure
OR: Odds ratio
HZO: Herpes zoster ophthalmicus
CMV: Cytomegalovirus
CD4: Cluster of differentiation 4 Helper T-Cell
PBS: Pharmaceutical Benefits Scheme
ABS: Australian Bureau of Statistics
ONH: Optic nerve head
RNFL: Retinal nerve fibre layer
OCT: Optical coherence tomography
VEP: Visual evoked potential
EREC: External Request Evaluation Committee
DHS: Department of Human Services
