# Supplementary material for: Increased incidence of glaucoma medication usage in middle-aged Australian males taking antiretroviral medication – a population-based study
Source: J Ophthalmic Inflamm Infect. 2020 Nov 3;10:30. doi: 10.1186/s12348-020-00218-y (PMC7609505; doi:10.1186/s12348-020-00218-y)
Supplement: Supplementary file 1 — Additional file 1. [file 12348_2020_218_MOESM1_ESM.docx]

# Supplementary Material

| Gender | Age group | N anti-HIV meds | N any PBS meds | Rate (95%CI) of anti-HIV medication, per 1000 people* |
| --- | --- | --- | --- | --- |
| Females | |  |  |  |
|  | 10 to 19 | 74 | 762502 | 0.10 (0.07, 0.12) |
|  | 20 to 29 | 607 | 940871 | 0.65 (0.59, 0.70) |
|  | 30 to 39 | 1612 | 1015032 | 1.59 (1.51, 1.67) |
|  | 40 to 49 | 1383 | 1049357 | 1.32 (1.25, 1.39) |
|  | 50 to 59 | 1073 | 1028244 | 1.04 (0.98, 1.11) |
|  | 60 to 69 | 601 | 1086520 | 0.55 (0.51, 0.60) |
|  | 70 to 79 | 219 | 683856 | 0.32 (0.28, 0.36) |
|  | 80 and above | 60 | 466152 | 0.13 (0.10, 0.16) |
|  |  |  |  |  |
|  | All ages (10 and older) | 5629 | 7032534 | 0.80 (0.78, 0.82) |
| Males | |  |  |  |
|  | 10 to 19 | 92 | 665153 | 0.14 (0.11, 0.17) |
|  | 20 to 29 | 1461 | 624879 | 2.34 (2.22, 2.46) |
|  | 30 to 39 | 3977 | 721274 | 5.51 (5.34, 5.69) |
|  | 40 to 49 | 6550 | 842447 | 7.77 (7.59, 7.96) |
|  | 50 to 59 | 6518 | 935144 | 6.97 (6.80, 7.14) |
|  | 60 to 69 | 3210 | 1005650 | 3.19 (3.08, 3.30) |
|  | 70 to 79 | 980 | 628596 | 1.56 (1.46, 1.66) |
|  | 80 and above | 126 | 300894 | 0.42 (0.35, 0.49) |
|  |  |  |  |  |
|  | All ages (10 and older) | 22914 | 5724037 | 4.00 (3.95, 4.05) |

**Table. Rate of anti-HIV medication prescriptions in the population for whom any PBS medication was prescribed (from July 2012 to Dec 2016, inclusive)**

| Gender | Age group | N anti-Glaucoma meds | N any PBS meds | Rate (95%CI) of anti-Glaucoma medication, per 1000 people* |
| --- | --- | --- | --- | --- |
| Females | |  |  |  |
|  | 10 to 19 | 675 | 762502 | 0.89 (0.82, 0.95) |
|  | 20 to 29 | 1494 | 940871 | 1.59 (1.51, 1.67) |
|  | 30 to 39 | 2936 | 1015032 | 2.89 (2.79, 3.00) |
|  | 40 to 49 | 7089 | 1049357 | 6.76 (6.60, 6.91) |
|  | 50 to 59 | 20022 | 1028244 | 19.47 (19.20, 19.74) |
|  | 60 to 69 | 51220 | 1086520 | 47.14 (46.73, 47.55) |
|  | 70 to 79 | 70907 | 683856 | 103.69 (102.92, 104.45) |
|  | 80 and above | 70966 | 466152 | 152.24 (151.12, 153.36) |
|  |  |  |  |  |
|  | All ages (10 and older) | 225309 | 7032534 | 32.04 (31.91, 32.17) |
| Males | |  |  |  |
|  | 10 to 19 | 852 | 665153 | 1.28 (1.19, 1.37) |
|  | 20 to 29 | 1324 | 624879 | 2.12 (2.00, 2.23) |
|  | 30 to 39 | 2692 | 721274 | 3.73 (3.59, 3.87) |
|  | 40 to 49 | 7124 | 842447 | 8.46 (8.26, 8.65) |
|  | 50 to 59 | 19606 | 935144 | 20.97 (20.67, 21.26) |
|  | 60 to 69 | 48409 | 1005650 | 48.14 (47.71, 48.57) |
|  | 70 to 79 | 66061 | 628596 | 105.09 (104.29, 105.89) |
|  | 80 and above | 48320 | 300894 | 160.59 (159.16, 162.02) |
|  |  |  |  |  |
|  | All ages (10 and older) | 194388 | 5724037 | 33.96 (33.81, 34.11) |

**Table. Rate per 1000 people of Glaucoma medication prescriptions in the population for whom any PBS medication was prescribed (from July 2012 to Dec 2016, inclusive)**
